# The Shift from Local to Global Visual Processing in 6-Year-Old Children Is Associated with Grey Matter Loss

**DOI:** 10.1371/journal.pone.0020879

**Published:** 2011-06-08

**Authors:** Nicolas Poirel, Grégory Simon, Mathieu Cassotti, Gaëlle Leroux, Guy Perchey, Céline Lanoë, Amélie Lubin, Marie-Renée Turbelin, Sandrine Rossi, Arlette Pineau, Olivier Houdé

**Affiliations:** 1 UMR 6232, CI-NAPS, CNRS, CEA, Caen University and Paris Descartes University, Sorbonne, France; 2 Centre de Gestion Scientifique, Mines ParisTech, Paris, France; 3 Centre Hospitalier Universitaire, Caen, France; 4 Institut Universitaire de France, Paris, France; Université Pierre et Marie Curie, France

## Abstract

**Background:**

A real-world visual scene consists of local elements (e.g. trees) that are arranged coherently into a global configuration (e.g. a forest). Children show psychological evolution from a preference for local visual information to an adult-like preference for global visual information, with the transition in visual preference occurring around 6 years of age. The brain regions involved in this shift in visual preference have not been described.

**Methods and Results:**

We used voxel-based morphometry (VBM) to study children during this developmental window to investigate changes in gray matter that underlie the shift from a bias for local to global visual information. Six-year-old children were assigned to groups according to their judgment on a global/local task. The first group included children who still presented with local visual processing biases, and the second group included children who showed global visual processing biases. VBM results indicated that compared to children with local visual processing biases, children with global visual processing biases had a loss of gray matter in the right occipital and parietal visuospatial areas.

**Conclusions:**

These anatomical findings are in agreement with previous findings in children with neurodevelopmental disorders and represent the first structural identification of brain regions that allow healthy children to develop a global perception of the visual world.

## Introduction

Recent evidences from magnetic resonance imaging (MRI) show that human brain development is characterized by nonlinear dynamic loss of gray mater (GM) with age that varies according to brain region [1], [2]. Even if the fine-tuning of GM is now well established, little is known about the relationship between brain structure variation and perception evolution in children [3]. Reduction in synaptic density, a phenomenon called “synaptic pruning,” is a fundamental neural plasticity mechanism that may underlie selective behavioral specialization [4]. The present study investigated selective specialization during a well-known developmental period in children in which the mode of visual perception changes.

**Figure 1:**
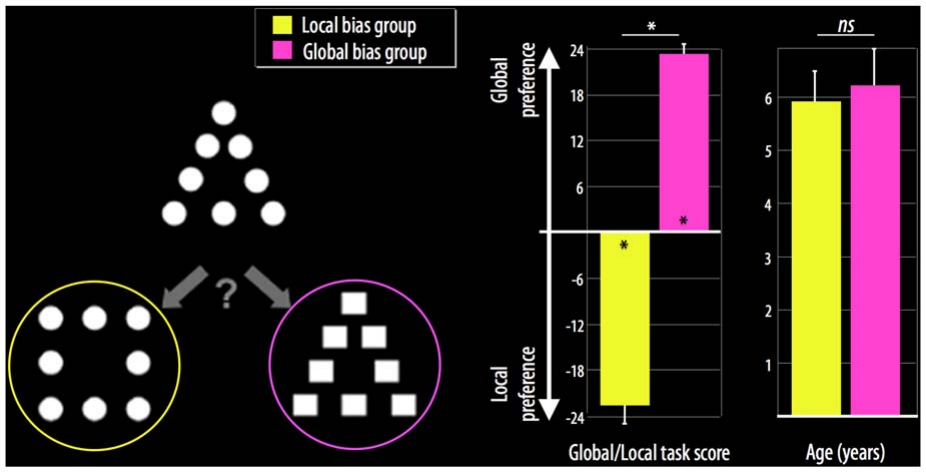
Representative example of a global/local triad stimulus (left), mean global/local task scores (middle), and mean age (right) for the local bias group (yellow) and the global bias group (pink). *p<0.05; ns = non-significant.

The visual world consists of local elements (e.g. trees) that are arranged coherently into a global configuration (e.g. a forest). Converging paradigms using compound stimuli (large global forms composed of arrangements of small local forms; see [5] and Fig. 1) clearly indicate an evolution from local preference (also called local bias) in young children to an adult-like global preference (also called global bias) by 9 years of age, with a transition occurring around 6 years of age [6], [7], [8]. This transition may be due to a shift in visuospatial strategy, i.e. a shift from a strategy of local sampling of visual information processing to an exhaustive adult-like global exploration of the visual stimuli [8], [9]. The brain regions allowing this shift in visual preference have not been identified, although neuropsychological and neuroimaging studies have indicated that different brain regions process global and local information. Adult patients with right hemisphere injuries show impaired processing of global level information, whereas patients with left hemisphere injuries present with deficits in processing local elements [10], [11]. These observations have been confirmed using functional imaging in healthy adults [12], [13] and in 14-year-olds [14]. Interestingly, children with perinatal brain lesions to the left or right hemispheres present with visuospatial deficits that mirror those in adult patients (see [15] for a review). Longitudinal studies of 5- to 12-year-old children by Stiles et al. also showed that overall, children with right perinatal lesions can accurately perceive local but not global elements of visual information, whereas children with left perinatal lesions show the reverse pattern. The authors also noted that although all of the children showed improved performance in terms of visual perception as they got older, the deficit pattern persisted for both groups. These studies revealed important information about the relationships between brain lesions and visuospatial development. However, the shift in bias from local to global visual processing that occurs around age 6 in healthy children has never been investigated.

The current study used voxel-based morphometry (VBM) of anatomical MRI images of children's brains to determine whether the shift from a local to a global visual processing bias corresponded to changes in gray matter. It has been proposed that the right hemisphere supports global information processing; thus, we expected that compared to children with a local visual processing bias (hereafter termed the “local bias” group), those with a global visual processing bias (the “global bias” group) would show GM loss mostly in right brain regions. This GM loss would represent selective brain specialization for global visual processing. More specifically, we expected to find GM loss in the right primary visual cortex and in the right lingual gyrus, areas that are strongly implicated in global processing in adults [12], [16]. Finally, the shift in visual processing bias might also induce GM loss in the right parietal regions [17]. Because the switch in visual preference concerns global visual processing, we did not expect differences between the two groups of children in the left hemisphere, which is involved in local visual processing, as noted above.

To test these hypotheses, we compared anatomical MRI images from 6-year-old children who presented with either a local or a global visual processing bias. In agreement with the principle of selective specialization, our hypothesis was that reduction in right hemisphere GM in children in the global bias group would be associated with the emergence of adult-like global visual perception.

## Methods

### Participants

Twenty-five children from Caen (Calvados, France) participated in this study (mean age, 6 years±1.6 months; 16 girls; 21 right handed children). The children had no history of neurological disease and no cerebral abnormalities as assessed by T1-weighted MRI. The local ethics committee (CPP Nord-Ouest III, France) approved the study. Written consent was obtained from the parents and the children themselves after detailed discussion and explanations.

### MRI acquisition and analysis

Anatomical images were acquired for each child on the same 3 T MRI scanner (Achieva, Philips Medical System, the Netherlands) using 3D T1-weighted spoiled gradient images (FOV: 256 mm; slice thickness: 1.33 mm; 128 slices; matrix size 192×192 voxels; 5 min 7 s duration). Brain images were acquired while the children passively watched a cartoon on an MRI-compatible screen. The sedative effects of the audio/visual system on children in MRI scanners have been demonstrated: specifically, this system reduces motion, provides a positive experience, and decreases wait times [18].

The T1 images were spatially normalized and segmented with SPM5 software (Welcome Department of Cognitive Neurology, www.fil.ion.ucl.ac.uk/spm) using a specific template built using the T1 images of our sample of children (the anatomical images were acquired with the same MRI scanner). A factorial VBM analysis [19] was performed using SPM5 software on normalized, modulated, and smoothed GM images by contrasting the two groups of children on the basis of their local/global scores (see below). This included a total brain volume correction for each subject.

### Local/global task

All children were presented with the global/local task at school after the laboratory MRI session [5], [20]. A total of 24 compound stimulus triads were presented to measure global/local bias in visual perception. Specifically, children judged which of two figures was most similar to a reference figure (Fig. 1). The judgment could be made based on either the local or global aspect of the reference. Children were instructed to give their first, most immediate similarity judgment for each trial. A measure of global/local precedence was calculated afterwards for each participant by subtracting the number of local choices from the number of global choices. The value range was −24 to 24, with a more positive value indicating a greater bias toward global visual information.

## Results

### Behavioral results

The children were grouped according to their scores on the local/global task. Children with negative scores were included in the local bias group, and children with positive scores were included in the global bias group (Fig. 1). In this sample, seven children showed a local visual processing bias (6 girls; 7 right-handed; mean score on the global/local task, −22.6±0.8) and 18 children showed a global visual processing bias (10 girls; 14 right-handed; mean score on the global/local task, 23.3±0.3). The global/local task scores differed significantly between the local bias group and the global bias group (*t*(23) = 67, *p*<0.0001). Importantly, the mean age was not significantly different for the two groups (*t*(23) = 0.99, *p* = 0.33; Fig. 1).

### VBM results

**Figure 2:**
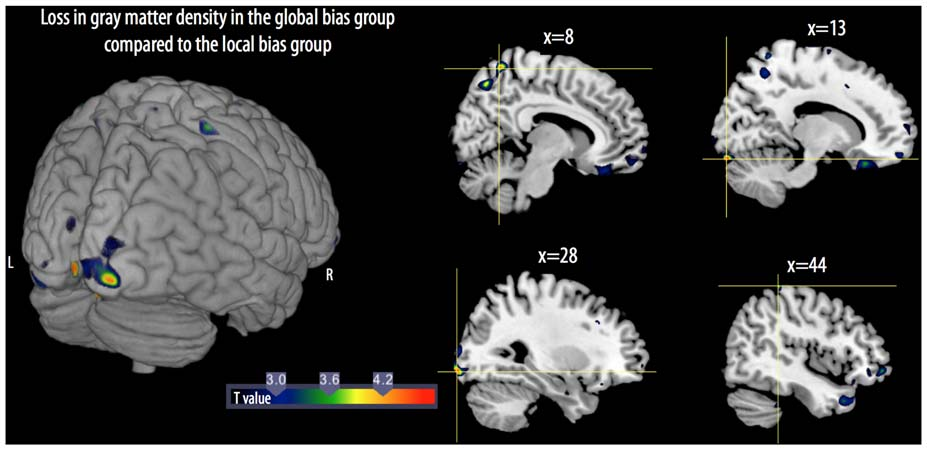
3D rendering (left) and sagittal views (right) show the loss of gray matter volume between the local bias group and global bias group of children. L: left; R: right. For illustrative purposes, the maps were thresholded at *p* = 0.01.

Contrast analyses were performed to identify changes in GM density between the two groups of children. GM variations were reported when voxels were significantly different at *p*<0.001 uncorrected, with a minimum of 50 voxels in clusters (Table 1). The contrast analysis between the two groups revealed that compared to the local bias group, the global bias group showed losses in GM in the occipital cortex, along the right part of the calcarine sulcus, the right inferior occipital gyrus extending to the middle occipital gyrus, and the lingual gyri (bilaterally) (Fig. 2). Note that the cluster in the lingual gyrus in the right hemisphere was larger than in the left hemisphere (279 *vs.* 58 voxels). Finally, GM loss was also observed in the parietal cortex, including losses in the right precuneus and the postcentral gyrus. The reverse comparison, i.e., subtraction of the GM density results of the local bias group from those of the global bias group revealed no significant differences.

**Table 1:** Anatomic localization, localization extent, MNI coordinates, and Z scores for maximal gray matter volume differences between the local bias group and the global bias group of children.

Anatomic localization	Number of voxels	Hemisphere	MNI coordinates			Z score
			X	Y	Z	
**Local bias ** ***minus*** ** Global bias**						
Calcarine	204	R	4	−100	−5	3.94
Lingual	279	R	13	−90	−19	3.93
Inf/Mid Occipital	278	R	28	−97	−8	3.83
		R	24	−102	−2	3.16
Precuneus	224	R	8	−56	65	3.73
	60	R	8	−67	52	3.56
Postcentral	56	R	44	−39	66	3.51
Lingual	58	L	−22	−71	−1	3.43
**Global bias ** ***minus*** ** Local bias**						
No significant difference						

L: left; R: right.

## Discussion

This study is the first to directly examine changes in GM density during the developmental window in childhood when there is a shift from a local visual processing bias to an adult-like global visual processing bias. In agreement with our previous findings, children at the transition age of 6 years presented either a local visual processing bias or an adult-like global visual processing bias [8]. Using VBM, we showed GM loss along the right calcarine sulcus in the global bias group of children compared to the local bias group of children, suggesting a fine-tuning of the primary visual cortex for processing global visual information. GM differences were also found in the right lingual gyrus and in the right parietal region.

In adults, the right middle occipital cortex is more activated during global tasks than during local tasks [16]. This early visual area is predominant during processing of natural visual scenes with low spatial frequency and is known to convey global information during visual processing [21]. At the same time, the right lingual gyrus is thought to function in global processing in adults [12]. Consequently, the GM loss in these regions that we observed in children may reflect selective specialization in the early stages of visual processing of global information. These results are in line with our recent data showing that the capturing of attention by global cues affects brain processing in the early visual stages processing [22]. The present study also revealed a GM loss in the right parietal cortex, which is involved in attentional focus toward global information [11], [21], [23]. More specifically, the GM variation in the precuneus may reflect the shift in attention to global rather than local features [24]. As was also shown in the present work, the postcentral gyrus is involved in global perception of coherent scenes [25]. Interestingly, repetitive transcranial magnetic stimulation (rTMS) findings indicate that the parietal cortex plays a key role in attention toward global information [26]. In particular, when the right posterior parietal cortex is stimulated by rTMS, the guidance of attention toward the salient global form of a stimulus is disrupted. Our results are clearly in agreement with the aforementioned roles of the parietal cortex. Taken together, the data showing loss of GM in the right parietal and visual areas in some 6-year-olds may reflect anatomical maturation processes that allow children to shift from a mode of local to global processing of visual information. Our results also suggest that a neurodevelopmental disorder of the dorsal stream, including in the right parietal and visual regions found in the present work, would create specific difficulties in processing global visual information. Recent neurodevelopmental data in individuals with Williams syndrome are in agreement with this idea [27]. Williams patients, usually defined as local spatial processors [28], present specifically reduced parietal and visual dorsal activation during global processing, whereas activation in the ventral occipito-temporal cortex is equivalent to controls. These results fit well with our findings that the emergence of a global visual preference in healthy children is accompanied by GM loss in the occipito-parietal dorsal pathway of the brain.

Finally, the present findings may provide a better understanding of some psychiatric disorders, such as schizophrenia. Indeed, global information processing is defective even in the early stages of perception in schizophrenia patients, resulting in a visual attraction toward the local properties of real-world scenes [29]. Difficulties in processing global information in schizophrenia patients are proposed to be due to impairment of the dorsal pathway [30]. According to the recent view that impairment in brain structure maturation is responsible, at least in part, for schizophrenia [31], the use of VBM with a focus on the brain regions identified in this study may be useful for exploring the neurodevelopmental origins of this pathology.

In conclusion, how we perceive the visual world as a coherent whole has been a central question in experimental psychology since the end of the 19^th^ century [32]. Since the end of the 20^th^ century, brain imaging techniques in adults and neuropsychological findings in children have given us a greater understanding of the neural basis of visual processing [12], [15]. The present study is the first to report specific brain regions that are involved in the perception of global visual information in healthy children.

## References

[1] Casey B, Tottenham N, Liston C, Durston S (2005). Imaging the developing brain: what have we learned about cognitive development?. Trends Cogn Sci.

[2] Shaw P, Kabani NJ, Lerch JP, Eckstrand K, Lenroot R (2008). Neurodevelopmental trajectories of the human cerebral cortex.. The Journal of Neuroscience: The Official Journal of the Society for Neuroscience.

[3] O'Hare E, Sowell E, Nelson DansA, Luciana M (2008). Imaging Developmental Changes in Gray and White Matter in the Human Brain.. Handbook of Developmental Cognitive Neuroscience (second edition, p. 23–38).

[4] Edelman GM (1993). Neural Darwinism: selection and reentrant signaling in higher brain function.. Neuron.

[5] Kimchi R (1992). Primacy of wholistic processing and global/local paradigm: a critical review.. Psychol Bull.

[6] Dukette D, Stiles J (2001). The effects of stimulus density on children's analysis of hierarchical patterns.. Developmental Science.

[7] Kimchi R, Hadad B, Behrmann M, Palmer S (2005). Microgenesis and ontogenesis of perceptual organization.. Psychol Sci.

[8] Poirel N, Mellet E, Houdé O, Pineau A (2008). First came the trees, then the forest: developmental changes during childhood in the processing of visual local-global patterns according to the meaningfulness of the stimuli.. Developmental Psychology.

[9] Vurpillot E (1968). The development of scanning strategies and their relation to visual differentiation.. Journal of Experimental Child Psychology.

[10] Delis D, Robertson L, Efron R (1986). Hemispheric specialization of memory for visual hierarchical stimuli.. Neuropsychologia.

[11] Robertson L, Lamb M (1991). Neuropsychological contributions to theories of part/whole organization.. Cognit Psychol.

[12] Fink G, Halligan P, Marshall J, Frith C, Frackowiak R (1996). Where in the brain does visual attention select the forest and the trees?. Nature.

[13] Martinez A, Moses P, Frank L, Buxton R, Wong E (1997). Hemispheric asymmetries in global and local processing: evidence from fMRI.. Neuroreport.

[14] Moses P, Roe K, Buxton R, Wong E, Frank L (2002). Functional MRI of global and local processing in children.. Neuroimage.

[15] Stiles J, Reilly J, Paul B, Moses P (2005). Cognitive development following early brain injury: evidence for neural adaptation.. Trends Cogn Sci.

[16] Han S, Weaver J, Murray S, Kang X, Yund E (2002). Hemispheric asymmetry in global/local processing: effects of stimulus position and spatial frequency.. Neuroimage.

[17] Weissman DH, Woldorff MG (2005). Hemispheric asymmetries for different components of global/local attention occur in distinct temporo-parietal loci.. Cerebral Cortex.

[18] Lemaire C, Moran GR, Swan H (2009). Impact of audio/visual systems on pediatric sedation in magnetic resonance imaging.. Journal of Magnetic Resonance Imaging.

[19] Ashburner J, Friston KJ (2000). Voxel-based morphometry-the methods.. Neuroimage.

[20] Kimchi R, Palmer S (1982). Form and texture in hierarchically constructed patterns.. Journal of Experimental Psychology : Human Perception and Performance.

[21] Peyrin C, Baciu M, Segebarth C, Marendaz C (2004). Cerebral regions and hemispheric specialization for processing spatial frequencies during natural scene recognition. An event-related fMRI study.. Neuroimage.

[22] Beaucousin V, Cassotti M, Simon G, Pineau A, Kotsova M (2011). ERP evidence of a meaningfulness impact on visual global/local processing: When meaning captures attention.. Neuropsychologia.

[23] Robertson L (1996). Attentional persistence for features of hierarchical patterns.. journal of Experimantal Psychology: General.

[24] Himmelbach M, Erb M, Klockgether T, Moskau S, Karnath H (2009). fMRI of global visual perception in simultanagnosia.. Neuropsychologia.

[25] Jung WH, Gu B, Kang D, Park J, Yoo SY (2009). BOLD response during visual perception of biological motion in obsessive-compulsive disorder: an fMRI study using the dynamic point-light animation paradigm.. European Archives of Psychiatry and Clinical Neuroscience.

[26] Mevorach C, Humphreys G, Shalev L (2006). Opposite biases in salience-based selection for the left and right posterior parietal cortex.. Nature Neuroscience.

[27] Mobbs D, Eckert MA, Menon V, Mills D, Korenberg J (2007). Reduced parietal and visual cortical activation during global processing in Williams syndrome.. Developmental Medicine and Child Neurology.

[28] Pani J, Mervis C, Robinson B (1999). Global spatial organization by individuals with Williams syndrome.. Psychol Sci.

[29] Poirel N, Brazo P, Turbelin MR, Lecardeur L, Simon G (2010). Meaningfulness and global-local processing in schizophrenia.. Neuropsychologia.

[30] Doniger G, Foxe J, Murray M, Higgins B, Javitt D (2002). Impaired visual object recognition and dorsal/ventral stream interaction in schizophrenia.. Archives of General Psychiatry.

[31] Rapoport JL, Gogtay N (2011). Childhood onset schizophrenia: support for a progressive neurodevelopmental disorder.. International Journal of Developmental Neuroscience.

[32] Koffka K (1935). Principles of Gestalt psychology.. New-York (Harcourt).

